# Prenatal Diagnosis of Recurrent Distal 1q21.1 Duplication in Three Fetuses With Ultrasound Anomalies

**DOI:** 10.3389/fgene.2018.00275

**Published:** 2018-08-20

**Authors:** Xiuqing Ji, Qiong Pan, Yan Wang, Yun Wu, Jing Zhou, An Liu, Fengchang Qiao, Dingyuan Ma, Ping Hu, Zhengfeng Xu

**Affiliations:** ^1^State Key Laboratory of Reproductive Medicine, Department of Prenatal Diagnosis, Affiliated Obstetrics and Gynecology Hospital with Nanjing Medical University, Nanjing Maternity and Child Health Care Hospital, Nanjing, China; ^2^Laboratory of Clinical Genetics, Department of Prenatal Diagnosis, Huai’an Maternal and Child Health Care Hospital, Huaian, China

**Keywords:** recurrent distal 1q21.1 duplication, prenatal diagnosis, chromosomal microarray analysis, nasal bone loss, duodenal atresia

## Abstract

**Background:** The phenotype of duplication of 1q21.1 region is variable, ranging from macrocephaly, autism spectrum disorder, congenital anomalies, to a normal phenotype. Few cases have been reported in the literature regarding prenatal diagnosis of 1q21.1 duplication syndrome. The current study presents prenatal diagnosis of 1q21.1 duplication syndrome in three fetuses with ultrasound anomalies.

**Case presentation:** Three fetuses from three unrelated families were included in the study. The prenatal routine ultrasound examination showed nasal bone loss in Fetus 1 and Fetus 3, as well as duodenal atresia in Fetus 2. Chromosomal microarray analysis was performed to provide genetic analysis of amniotic fluid and parental blood samples. The CMA results revealed two *de novo* duplications of 1.34 and 2.69 Mb at distal 1q21.1 region in two fetuses with absent nasal bone, as well as a maternal inherited 1.35-Mb duplication at distal 1q21.1 in one fetus with duodenal atresia.

**Conclusions:** The phenotype of 1q21.1 duplication syndrome in prenatal diagnosis is variable. The fetuses with nasal bone loss or duodenal atresia may be related to 1q21.1 duplication and chromosomal microarray analysis should be performed.

## Background

Individuals with 1q21.1 duplication (OMIM 612475) are reportedly associated with a spectrum of macrocephaly, autism spectrum disorder, dysmorphic features, and congenital anomalies. In recent years, the application of CMA allows for more patients with 1q21.1 duplication to be revealed (Brunetti-Pierri et al., 2008; Mefford et al., 2008; Rosenfeld et al., 2012; Soemedi et al., 2012; Dolcetti et al., 2013; Verhagen et al., 2015; Bernier et al., 2016; Buse et al., 2017; Wang et al., 2017). Highly diverse inter- and intrafamilial outcomes were observed among patients harboring 1q21.1 duplication, from almost normal to severely affected (Brunetti-Pierri et al., 2008; Mefford et al., 2008; Dolcetti et al., 2013; Buse et al., 2017).

Chromosomal region 1q21.1 is structurally complex with many SDs that make it prone to NAHR (Sharp et al., 2005; Mefford et al., 2008). Some scholars believed that the chromosome 1q21.1 region can be subdivided into two distinctive regions (Rosenfeld et al., 2012; Verhagen et al., 2015; Wang et al., 2017). The proximal region, which is flanked by SDs, extends recurrent BPs from BP2 and BP3 (spans about 200 Kb), and the distal region extends from BP3 to BP4 (spans about 1.35 Mb). Individuals with the proximal duplication had variable phenotypic features, with the most common features: intellectual disability (ID), dysmorphic features, and behavioral problem (Rosenfeld et al., 2012). More patients with distal duplication were reported and manifested ID, autism, congenital heart disease (CHD) (e.g., tetralogy of Fallot), macrocephaly, and mild dysmorphic features (e.g., frontal bossing and hypertelorism) (Brunetti-Pierri et al., 2008; Mefford et al., 2008; Rosenfeld et al., 2012; Dolcetti et al., 2013).

Due to the limited number of cases being reported, the incidence of this disease has not been reported in the population. Brunetti-Pierri et al. (2008) reported that 1q21.1 duplication was detected in 17 out of 16557 (0.103%) patients from a wide range of referring diagnoses, including mental retardation, autism and/or congenital anomalies. Mefford et al. (2008) identified nine persons with a recurrent 1.35-Mb duplication of 1q21.1 in 5218 (0.172%) patients with mental retardation, autism spectrum disorder and other variable features. Duplications at 1q21.1 were rare in control populations, with an incidence of 0.027% (Soemedi et al., 2012; Dolcetti et al., 2013).

Up to now, only two prenatal cases with 1q21.1 duplication were reported in literature (Liao et al., 2012; Verhagen et al., 2015). To provide a better understanding of this submicroscopic imbalance in prenatal diagnosis, we present our study on prenatal diagnosis of recurrent distal 1q21.1 duplication in three fetuses with ultrasound anomalies.

## Case Presentation

**FIGURE 1:**
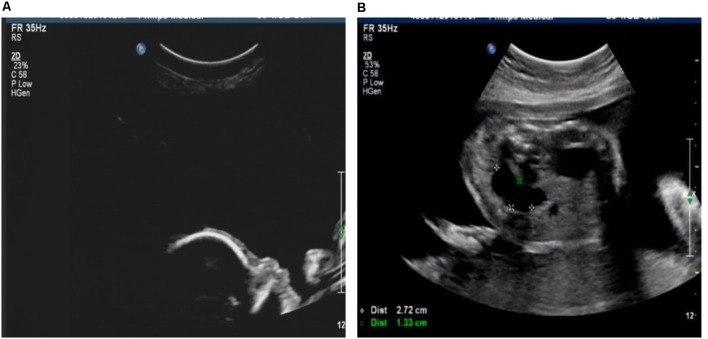
**(A)** Fetus with absent nasal bone. **(B)** Fetus with duodenal atresia.

**FIGURE 2:**
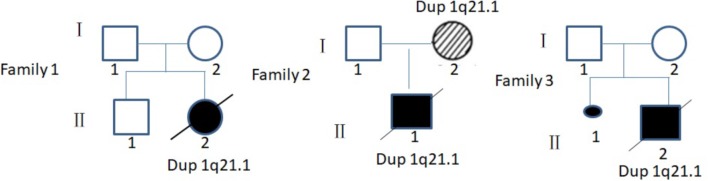
Pedigree of three families in this study. I, parent; II, offspring; 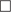, male; 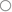, female; 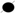, induced labor female fetus; 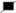, induced labor male fetus; 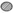, dup 21.1 patient without clinical symptoms; 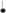, aborted fetus.

Case 1 was a fetus with absent fetal nasal bone detected by routine ultrasound examination during the second trimester (**Figure 1A**). Down’s screening was at high risk of chromosome 21 (1/215). After genetic counseling, amniocentesis was performed at 24 weeks of gestation. The parents were non-consanguineous and parental blood samples were drawn. Family history was unremarkable. Case 2 was a fetus identified with duodenal atresia during the second trimester ultrasound examination (**Figure 1B**). The rest of the fetal morphology was normal. After genetic counseling, amniotic fluid sample was obtained at 25 weeks of gestation and parental blood samples were drawn. The mother’s phenotype is completely normal and she had no significant medical, surgical, or family history. Case 3 was a fetus identified with nasal bone absence by prenatal ultrasound morphology scan. After genetic counseling, amniotic fluid sample was collected at 26 weeks. Family history was unremarkable. Parental blood samples were collected. Clinical data of three fetuses are listed in **Table 1** and **Figure 2**.

**Table 1:** Summary of clinical data and genomic information on three fetuse.

	Fetus 1	Fetus 2	Fetus 3
Gestational week	24+	25+	26+
Age of mother	30	28	23
Gravida and para	G2P1	G1P0	G2P0
Ultrasound manifestation	Absent nasal bone	Duodenal atresia	Absent nasal bone
Karyotype	46,XX	46,XY	46,XY,
Results of CMA [hg19]	arr1q21.1q21.2 (146,476,526-147,820,342) × 3	arr1q21.1q21.2 (146,476,526-147,826,789) × 3	arr1q21.1q21.2 (146,510,112-149,205,098) × 3
Genetic mode of inheritance	*De novo*	Maternal	*De novo*


Amniotic fluid cell sample and peripheral blood of the parents were collected for cell culture followed by karyotyping. GTG-banding was performed according to a standard protocol. Karyotypes were determined from G-banding analysis using standard protocol according to the ISCN 2009 nomenclature.

Genomic DNA was extracted from uncultured amniotic fluid or blood cells, using the QIAamp DNA Mini Kit (QIAGEN, Hilden, Germany). Genomic DNA was examined by Human cyto12 SNP-array scanning (Illumina, United States), which comprised about 300,000 SNPs across the whole genome. SNP-array experiments were carried out as previously described (Hu et al., 2013) and molecular karyotype analysis was performed by KaryoStudio V 1.3.11(Illumina). Parental blood samples from each fetus were also obtained for the microarray analysis.

**FIGURE 3:**
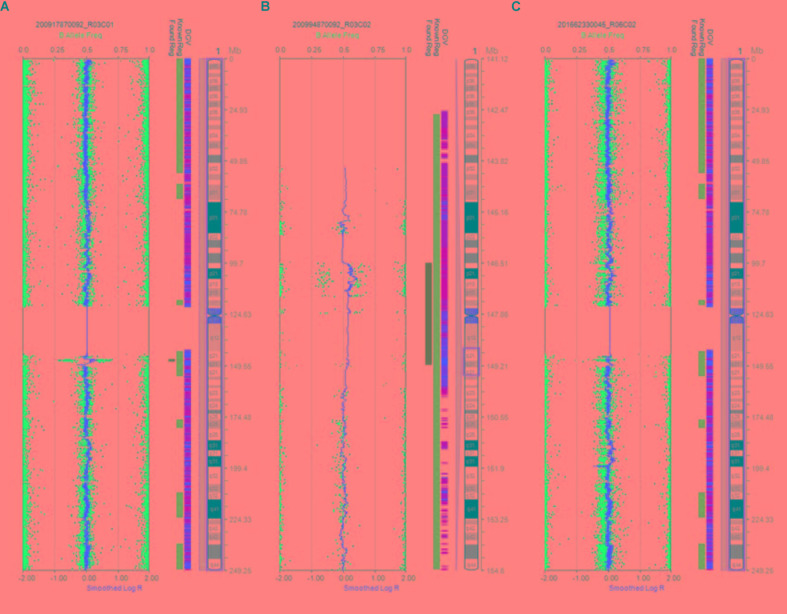
Results of CMA in three 1q21.1 duplication fetuses. **(A)** The original version. **(B)** The enlarged version. **(C)** Normal chromosome 1.

**FIGURE 4:**
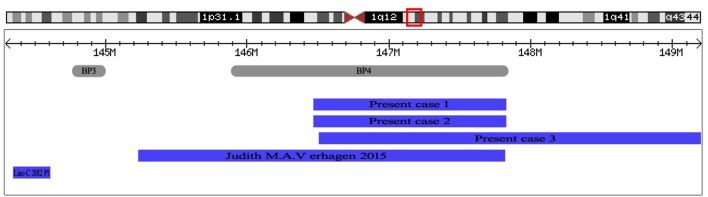
The positions of 1q21.1 duplication cases reported in prenatal diagnosis. At the top of the figure is an ideogram showing chromosome band 1q21.1 with genomic coordinates corresponding to the hg19 build of the human genome. Gray bars represent clusters of SD blocks BP3 and BP4 that contain the BPs of the recurrent rearrangements. Plots (blue shading) show the recurrent 1q21.1 duplications found in subjects reported in prenatal diagnosis.

Karyotype analysis showed that all the fetuses and their parents had normal karyotype. The CMA results of Fetus 1 and the parents revealed a *de novo* 1.34-Mb duplication at 1q21.1q21.2 containing nine OMIM genes (chromosome position: 146476526–147820342). The CMA results of Fetus 2 and the parents revealed a maternal inherited 1.35-Mb duplication at 1q21.1q21.2 encompassing nine OMIM genes (chromosome position: 146476526–147826789). The CMA results of Fetus 3 and the parents revealed a *de novo* 2.69-Mb duplication at 1q21.1q21.2 encompassing 14 OMIM genes (chromosome position: 146510112–149205098) (**Table 1** and **Figures 3**, **4**). We offered detailed genetic counseling to the couples and informed them about the variable phenotypes of the 1q21.1 duplication syndrome. All the three couples ultimately chose to terminate the pregnancies.

## Discussion and Conclusions

In this study, we report three prenatal cases of distal 1q21.1 duplication syndrome. The results of CMA revealed two *de novo* duplications of 1.34 Mb and 2.69 Mb at distal 1q21.1 in Fetus 1 and Fetus 3 with absent nasal bone, respectively, and a maternal inherited 1.35-Mb 1q21.1 duplication in Fetus 2 with duodenal atresia.

Phenotypic features described in association with 1q21.1 duplication are highly variable (Brunetti-Pierri et al., 2008; Mefford et al., 2008; Verhagen et al., 2015). Many patients with the 1q21.1 duplication have been described, yet only two cases were reported in prenatal setting. Verhagen et al. (2015) reported a fetus with distal 1q21.1 duplication (2.6 Mb, 145243316–147814694, **Figure 4**) with dilated ventricles, a ventricular septal defect, and dilated main pulmonary artery and aorta. Liao et al. (2012) described a fetus with proximal 1q21.1 duplication (258 Kb, 144337316–144595988, **Figure 4**) with bilateral polycystic kidney, oligohydramnios, ventricular septal defect. In this study, we describe three fetuses with distal 1q21.1 duplication spanning from BP3 to BP4 in the prenatal diagnosis. Fetus 1 and Fetus 3 with absent nasal bone exhibited 1q21.1 duplication of 1.34 Mb (146476526–147820342) and 2.69 Mb (146510112–149205098), respectively. Fetus 2 with distal 1q21.1 duplication (1.35 Mb, 146476526–147826789) exhibited the feature of duodenal atresia.

Nasal bone absence is an important marker during the first or second trimester in prenatal screening for trisomy 21 (Sonek et al., 2006; Persico et al., 2012). The incidence of trisomy 21 in fetuses with absent or hypoplastic nasal bone varied widely, ranging from 26 to 77% (Moreno-Cid et al., 2014). A few cases with absent nasal bone were reported to be associated with the CNV. Brisset et al. (2015) reported duplication of 1q12q21.2 of 5.8 Mb associated with deletion of 16p11.2 of 545 Kb in a fetus exhibiting an absent nasal bone. Chen et al. (2017) reported an absent nasal bone fetus with a 2.752-Mb duplication at 4q11, a 1.949-Mb duplication at 4q13.2, and a 1.65-Mb deletion at 5q13.2, exhibiting no other apparent phenotypic abnormality. In our study, two 1q21.1 duplication fetuses displayed absent nasal bone. Therefore, the phenotype is not only related to Down’s syndrome, but also associated with CNV. It is suggested that CMA may be offered to fetuses with absent nasal bone in prenatal diagnosis.

The long-term prognosis of fetal digestive tract malformation depends on whether it is accompanied by chromosomal abnormalities or whether it has other malformations (Stoll et al., 2015). The typical double bubble sign, which strongly indicated duodenal atresia (Pariente et al., 2012), was found in Fetus 2 in our study. When duodenal atresia is detected prenatally, it is usually associated with a high incidence of trisomy 21 (Choudhry et al., 2009; Yang et al., 2017). Stoll et al. (2015) summarized that in the 728 cases of trisomy 21, 216 cases (30%) were identified with duodenal atresia. However, duodenal atresia has not been reported in cases of 1q21.1 duplication previously. This is the first prenatal case of distal 1q21.1 duplication exhibiting duodenal atresia.

The overlapping region of the recurrent 1q21.1 duplication in the three fetuses included nine OMIM genes: *PRKAB2*, *FMO5*, *CHD1L*, *BCL9*, *ACP6*, *GJA5*, *GJA8*, *NBPF24*, and *GPR89B*. The abnormal expression of *GJA5* and its flanking gene *GJA8* have been previously reported to be associated with CHD (Soemedi et al., 2012; Dolcetti et al., 2013; Verhagen et al., 2015). Dou et al. (2017) speculated that *CHD1L* can promote neuronal differentiation in hESCs and play an important role in nervous system development. Kimura et al. (2015) explored *BCL9* gene, and suggested it may be a susceptibility gene of schizophrenia. The two genes might associate with nervous system problems in 1q21.1 duplication of postnatal patients. Nevertheless, no genes within the overlapping region were found to be associated with duodenal atresia.

Many postnatal cases with 1q21.1 duplication have been identified with mental retardation, autism, multiple congenital anomalies, and other psychiatric disorders. A subset of individuals is clinically unaffected (Brunetti-Pierri et al., 2008; Mefford et al., 2008; Dolcetti et al., 2013; Buse et al., 2017). In our study, the 1q21.1 duplication in Fetus 1 and Fetus 3 occurred *de novo*, and Fetus 2 was maternal inherited. The penetrance for distal 1q21.1 duplication was reported to be 29.1% (Rosenfeld et al., 2013). The incomplete penetrance and variable expressivity of the duplication syndrome pose challenges for counseling regarding prenatal diagnoses, especially for the syndrome associated with neurodevelopmental phenotypes that cannot be ascertained prenatally. Therefore, in the prenatal genetic counseling session, comprehensive and detailed information about the estimates for penetrance and the range of possible phenotypic outcomes of the syndrome should be offered to the prospective parents. In this study, after comprehensive genetic counseling, the couples ultimately chose to terminate the pregnancies. Routine ultrasound screening during the second trimester of pregnancy is performed between 22 and 28 weeks of gestation in China. Therefore, amniocentesis was carried out late in these cases. In China, it is legal to induce labor before 28 weeks of gestation if the fetus is identified to be with severe structural malformation or pathogenic chromosomal abnormalities. This study is a retrospective study, so it is regretful that the pregnant women and their families have refused the autopsy. However, we carried out the follow-up study and no obvious abnormalities were observed in the appearance of the three fetuses after termination of the pregnancies.

In summary, we reported the prenatal diagnosis of distal 1q21.1 duplication in three fetuses with absent nasal bone or duodenal atresia. The phenotype of 1q21.1 duplication is highly variable prenatally, like postnatally. Combined with ultrasonic examination, the application of CMA will improve the diagnosis of recurrent rearrangements of 1q21.1 in fetuses. Identification of additional affected fetuses with similar rearrangements of chromosome 1q21.1 is needed to provide further insights into the pathogenesis of 1q21.1 duplication syndrome.

## Ethics Statement

The study was reviewed and approved by the ethical committee of the Affiliated Obstetrics and Gynecology Hospital with Nanjing Medical University in China. Written informed consent to participate was obtained for all participants or their guardians before collecting samples of amniotic fluid and blood. Written informed consent was obtained from the patient for publication of this research.

## Availability of Data and Materials

All data generated or analyzed during this study are included in this published article.

## Author Contributions

XJ and QP wrote the paper and carried out molecular genetic studies. ZX and PH helped to draft the manuscript. YaW and JZ conducted genetic counseling. YuW conducted ultrasonic testing. AL, FQ, and DM carried out the laboratory work. All authors read and approved the final manuscript.

## Conflict of Interest Statement

The authors declare that the research was conducted in the absence of any commercial or financial relationships that could be construed as a potential conflict of interest. The reviewer IRH and handling Editor declared their shared affiliation.

## Abbreviations

BPs: breakpoints
CMA: chromosomal microarray analysis
CNV: copy number variant
NAHR: non-allelic homologous recombination
SDs: segmental duplications
